# A 3-dimensional human embryonic stem cell (hESC)-derived model to detect developmental neurotoxicity of nanoparticles

**DOI:** 10.1007/s00204-012-0984-2

**Published:** 2012-12-02

**Authors:** Lisa Hoelting, Benjamin Scheinhardt, Olesja Bondarenko, Stefan Schildknecht, Marion Kapitza, Vivek Tanavde, Betty Tan, Qian Yi Lee, Stefan Mecking, Marcel Leist, Suzanne Kadereit

**Affiliations:** 1 Department of Biology, University of Konstanz, Universitaetsstrasse 10, 78457 Konstanz, Germany; 2 Konstanz Research School Chemical Biology, University of Konstanz, Konstanz, Germany; 3 Department of Chemistry, University of Konstanz, Konstanz, Germany; 4 Laboratory of Molecular Genetics, National Institute of Chemical Physics and Biophysics, Tallinn, Estonia; 5 Bioinformatics Institute Agency for Science Technology and Research (A*STAR), Singapore, Singapore; 6 Institute for Medical Biology, A*STAR, Singapore, Singapore

**Keywords:** Human embryonic stem cells, Neurospheres, Developmental neurotoxicity (DNT), Polyethylene nanoparticles, Methylmercury

## Abstract

Nanoparticles (NPs) have been shown to accumulate in organs, cross the blood–brain barrier and placenta, and have the potential to elicit developmental neurotoxicity (DNT). Here, we developed a human embryonic stem cell (hESC)-derived 3-dimensional (3-D) in vitro model that allows for testing of potential developmental neurotoxicants. Early central nervous system PAX6^+^ precursor cells were generated from hESCs and differentiated further within 3-D structures. The 3-D model was characterized for neural marker expression revealing robust differentiation toward neuronal precursor cells, and gene expression profiling suggested a predominantly forebrain-like development. Altered neural gene expression due to exposure to non-cytotoxic concentrations of the known developmental neurotoxicant, methylmercury, indicated that the 3-D model could detect DNT. To test for specific toxicity of NPs, chemically inert polyethylene NPs (PE-NPs) were chosen. They penetrated deep into the 3-D structures and impacted gene expression at non-cytotoxic concentrations. NOTCH pathway genes such as HES5 and NOTCH1 were reduced in expression, as well as downstream neuronal precursor genes such as NEUROD1 and ASCL1. FOXG1, a patterning marker, was also reduced. As loss of function of these genes results in severe nervous system impairments in mice, our data suggest that the 3-D hESC-derived model could be used to test for Nano-DNT.

**Electronic supplementary material:**

The online version of this article (doi:10.1007/s00204-012-0984-2) contains supplementary material, which is available to authorized users.

## Introduction

Engineered nanoparticles (NPs) are incorporated into an increasing number of commercial products, ranging from food constituents and cosmetics to electronics, coatings, paints and optics, and are explored for medical applications, and soil and water remediation. It can thus be expected that human exposure will increase. NPs have been shown to be toxic in vivo in animal models and in vitro cell cultures (Arora et al. 2012). A wealth of data demonstrates that NPs enter the blood circulation and organs, including the brain, and cross the placenta. This points toward potential health risks for humans, including cardiovascular disease, pulmonary diseases, impairment of brain function and developmental toxicity (Buzea et al. 2007; Wick et al. 2010). Mechanisms of toxicity are diverse and include oxidative stress, integration into mitochondria, activation of immune responses, changes in receptor or channel function by incorporated NPs, and interaction with enzymes. Toxicity mechanisms differ between the different NPs and depend on their chemical composition, shape, and surface properties (Buzea et al. 2007).

Developmental neurotoxicity (DNT), that is, impairment of nervous system development, with resulting structural or functional defects is difficult to model in animals. The potential of NPs to cause DNT is suggested by the finding that prenatal exposure to low concentrations of diesel exhaust containing NPs affected locomotor activity and the monoaminergic system in mice (Suzuki et al. 2010). Furthermore, studies have detected behavioral changes and alterations in gene expression in the brain of rodents after prenatal exposure to titanium dioxide (TiO_2_) NPs. Genes associated with apoptosis, oxidative stress, brain development, and psychiatric disease were altered (Hougaard et al. 2010; Shimizu et al. 2009). Therefore, there is an urgent need to assess the potential of engineered NPs to elicit DNT in humans.

Embryonic stem cells (ESC) have been shown to faithfully recapitulate stages of early neural development and are increasingly used to investigate neural development and to assess DNT (Colleoni et al. 2011; Stummann et al. 2009; Zimmer et al. 2011b, 2012). Here, we developed a three-dimensional (3-D) in vitro model derived from human embryonic stem cells (hESCs) to evaluate DNT of chemically inert polyethylene NPs (PE-NPs). A 3-D model has the advantage to provide an environment to the differentiating cells that allows for 3-D cellular interactions, similar to the in vivo situation where developing cells are exposed to 3-D signals and morphogen gradients. Exposure to the known developmental neurotoxicant, methylmercury, indicated sensitivity of the model. When exposing the model to non-cytotoxic concentrations of PE-NPs, we measured a reduction in the expression of neural markers, suggesting that the 3-D model could be used to assess NP-induced DNT (Nano-DNT).

## Materials and methods

### Cells and differentiation cultures

Human embryonic stem cells (hESCs, WA09 line) were obtained from WiCell (Madison, WI, USA) and cultured according to standard protocol (Thomson et al. 1998). Import of hESCs and experiments described herein are approved under license # 1710-79-1-4-27. WA09 cells were differentiated in adherent culture to PAX6^+^ neural progenitor cells as described, with slight modifications (Chambers et al. 2009). Briefly, differentiation was initiated on day 3 (labeled ‘hESC’ in figures) by replating WA09 cells in single-cell suspension onto Matrigel-coated (BD Biosciences, Franklin Lakes, NJ USA) plates. Three days later, on day 0 of differentiation (d0), neural differentiation was promoted by adding neural differentiation medium and dual SMAD inhibition. On day 8 (d8), the adherent cells were digested to small clumps with dispase (Invitrogen, Carlsbad, USA) and transferred to low-adhesion plates (Corning, Corning, USA) in DMEM/F12 medium supplemented with B27 (Invitrogen, Carlsbad, USA), noggin (42 ng/ml; R&D Systems Minneapolis, USA), dorsomorphin (600 nM, Tocris Bioscience, Bristol, UK), FGF2 (20 ng/ml, R&D Systems Minneapolis, USA), and 10 μM ROCK inhibitor (Tocris, Bristol, UK). After three days, the medium was carefully aspirated and replaced by fresh medium without ROCK inhibitor. Every 3–4 days, medium was changed and neurospheres were sheered slightly to prevent large aggregations.

### Transcriptome analyses

mRNA was prepared from replicate (>6) cultures on indicated days from undifferentiated hESCs (day 3), d0, d5, d15, d22, and d25 cells. Replicates were pooled, and RNA processing and probe preparation were preformed as described (Zimmer et al. 2011a). For hESCs, d0, and d5 of differentiation, biological triplicates were generated. For d15 biological duplicates, and for d22 and d25 of differentiation, biological singletons were produced. Each sample was hybridized in triplicates to Illumina Sentrix HumanHT-12 BeadChip gene arrays (Illumina, San Diego, USA). Technical replicates were assigned to each array using a block randomized design subject to the constraint that each replicate was run on at least two different BeadChips to eliminate batch effects. The BeadChips were scanned and data were acquired using BeadStudio software. The average signal intensity values were normalized using quantile normalization in BeadStudio. Further statistical analyses and data analyses (principal component analysis (PCA), gene ontology (GO), hierarchical, and self-organizing map (SOM) clustering were performed with Partek Genomic Suite software (Partek, Inc., MI, USA) with a false discovery rate (FDR) of 0.01 or 0.005 to identify genes that changed significantly during the differentiation process. FDR was calculated using the Benjamini–Hochberg method. Hierarchical and SOM clustering were unsupervised. The raw microarray data are deposited in the EBI array express database (accession number E-MTAB-1343).

### Immunofluorescence and flow cytometry

Cells were fixed and stained with standard methods as described (Zimmer et al. 2011b), using the following antibodies: PAX6 (Covance, Princeton, New Jersey), NES (R&D Systems, Minneapolis, USA), A2B5, PSA-NCAM, CD133 (Miltenyi Biotech, Bergisch Gladbach, Germany), and CXCR4 (BD Biosciences, Franklin Lakes, NJ USA). For flow cytometry, single-cell suspensions were prepared on indicated days of differentiation by accutase (PAA, Pasching Austria) digest and stained as described (Kadereit et al. 2002). Fluorescence was acquired on an Accuri flow cytometer C6 (BD Biosciences, Franklin Lakes, NJ USA) and analyzed with CFlow software (BD Biosciences, Franklin Lakes, NJ USA). For the incorporation of PE-NPs, single-cell suspensions were prepared after 24 and 48 h of exposure, and fluorescence emitted by the PE-NP was acquired on an Aria flow cytometer (BD Biosciences, Franklin Lakes, NJ USA) and analyzed with Diva software (BD Biosciences, Franklin Lakes, NJ USA).

### Quantitative RT-PCR

mRNA was extracted at indicated time points from pooled duplicate culture wells and processed as described (Zimmer et al. 2011a). Real-time quantification for each gene was expressed relative to the amount of the housekeeping gene RPL13A for expression kinetics and PE-NP toxicity experiments, and the geometric mean of RPL13A and GAPDH for methylmercury toxicity experiments (Vandesompele et al. 2002). Expression was calculated using the 2^(−delta delta C(t)) method. The list of primers is given in Suppl. 1.

### Toxicity testing

All toxicity experiments on neurospheres started on day three after initiation of suspension cultures (d11). Neurospheres were treated either for 48 h in acute testing or for 18 days in DNT testing. For DNT, toxicants (methylmercury or PE-NPs) were replenished with each medium change. LUHMES cells (human neuronal precursors) were cultured and differentiated as described (Schildknecht et al. 2009) for 4 days to neurons and then treated for 48 h with PE-NPs at the indicated concentrations. As NPs at high concentrations interfered with the resazurin reduction assay, cell viability was indirectly assessed by measuring intracellular ATP content as an alternative endpoint for cell viability (Schildknecht et al. 2009). Otherwise, cell viability was assessed by standard resazurin reduction assay (Zimmer et al. 2011b).

### Polyethylene nanoparticle synthesis

Polyethylene nanoparticles (PE-NPs) were prepared in an aqueous microemulsion process, yielding narrowly dispersed anisotropic nanocrystals with a number average particle size of typically 33 nm. Between different batches, this average varied by 2 nm. For reduction of the high surfactant (sodium dodecyl sulfate) content necessary in this procedure, the as-obtained polymer dispersions were extensively dialyzed against water to yield dispersions with a typical surfactant and polymer content of <0.2 and >1 %, respectively, and a zeta potential of −30 mV. The high surface tension of >65 mN m^−1^ indicated complete removal of free surfactant. However, colloidal stability of the nanoparticles in culture medium was fully retained, as demonstrated by dynamic light scattering (DLS) measurements (see Suppl. 2). As control for synthesis residues, the aqueous PE-NP dispersions were centrifuged with Macrosep Advance centrifugation units (10 kDa cut-off), to filter out the NPs from the solvent. This NP-free solution (‘solvent’) was then used as a negative control, compared to the non-centrifuged PE-NP dispersions. Fluorescently labeled NPs were synthesized as above, while incorporating a perylene diimide (PDI) dye as fluorescence marker. Covalent linking of the dye prevented phase separation and undesired release of the dye from the nanoparticles. For more details, see Suppl. 2.

### Statistics

All experiments were performed with at least three biological replicates. Quantitative RT-PCR experiments were performed in technical duplicates or triplicates. To evaluate significant changes in gene expression compared to control cells, paired t-tests were performed in GraphPad Prism software using the log-transformed delta Ct-values to determine the significance of changes in gene expression between the mean delta Ct-values of untreated cells and methylmercury-treated cells, or between solvent and PE-NPs-treated cells.

## Results

### Differentiation of hESCs along the neural lineage

**Fig. 1:**
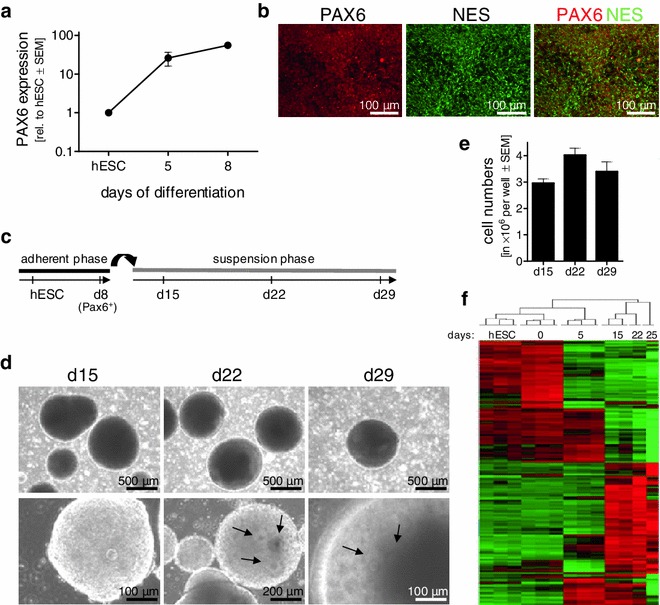
Differentiation of hESCs-derived PAX6^+^ cells to neural progenitors in a three-dimensional neurosphere system. hESCs were differentiated for 8 days in monolayer cultures to highly enriched Pax6^+^ cells. On day 8 (d8), cells were detached and replated as small clumps into suspension cultures to mature further for additional 21 days (d08–29). **a** On indicated days, mRNA was prepared and analyzed for the expression of PAX6 by qPCR. PAX6 expression in hESCs was set arbitrarily to 1 and expression calculated relative to expression in hESCs. Mean values ± SEM, *n* = 3. **b** PAX6 (*red*) and nestin (NES) (*green*) staining of adherent cells on d8. **c** Culture scheme to differentiate PAX6^+^ cells further within neurospheres. **d** Phase contrast images of neurospheres on days of differentiation 15, 22, and 29 (d15, d22, d29). Lower panels show higher magnifications of neurospheres with visible rosettes (*arrows*). **e** On indicated days, single-cell suspensions were made from neurosphere cultures and cell numbers counted per well. Shown are counts of 6–10 biological replicates (mean ± SEM). **f** Hierarchical clustering analysis of genes significantly regulated (*p* < 0.01) during neural differentiation of hESCs to day 25 cells

To model the three-dimensional (3-D) situation of early human central nervous system (CNS) development, we developed a hESC-derived culture system in which CNS progenitor cells mature in a 3-D neurosphere system. We first differentiated hESCs to an almost pure population of CNS PAX6^+^ progenitor cells as described (Chambers et al. 2009). Monitoring PAX6 expression by quantitative real-time PCR revealed peak expression on day 8 (d8) of differentiation (Fig. 1a). On d8, cells expressed the neural stem cell marker NES (nestin) and were highly enriched for PAX6 protein-expressing cells (Fig. 1b). As further differentiation of PAX6^+^ cells in adherence was inefficient (data not shown), we detached the cells on d8 and replated them as small clumps into suspension culture (Fig. 1c). Within 24 h, the cells formed round spheres (neurospheres). With increasing differentiation time, rosette-like structures became visible within the neurospheres (Fig. 1d, lower panels, arrows), suggesting progressing differentiation. During the differentiation process, cell numbers per well were reproducible between experiments (Fig. 1e).

### Transcriptome profiling during differentiation

To characterize the differentiation process within the 3-D neurosphere structures in more detail, mRNA was prepared and transcriptomes of the differentiating cells were analyzed with microarrays during the differentiation process. Notably, the transcriptomes of each differentiation stage differed significantly from each other and clustered readily apart, with large blocks of genes progressively downregulated, as well as blocks of genes that were upregulated over time, indicative of progressive changes in gene expression patterns during differentiation (Fig. 1f, Suppl. 3a). Interestingly, among the top 30 regulated genes, 21 increased during differentiation (Suppl. 4). Among these, marker genes for neural and neuronal development such as DMRT3 (a transcription factor expressed in the developing forebrain), RSPO1 (Wnt signaling), GPM6A (neuronal membrane glycoprotein), and TRH (tri-peptide neurotransmitter and neuromodulator in both central nervous and peripheral systems) were particularly strongly upregulated. Genes central to neurodevelopment were also upregulated (Table 1). High expression of patterning markers such as PAX6, EMX2, and LHX2 suggested the generation of cerebral cortex precursor cells (Monuki et al. 2001; Muzio and Mallamaci 2003). Pluripotency factors, on the other hand, such as POU5F1, NANOG, UTF1, LEFTY1, DPPA4 and CD24 were strongly downregulated during differentiation (Suppl. 5, 6).

**Table 1:** Neurodevelopmental genes significantly upregulated during differentiation within neurospheres

Gene		*p* value	
DLL1	Delta like	5.57E−04	Neural precursor
HES5	Transcriptional repressor	2.26E−04	Neural precursor
ASCL1	Mash1, achaete-scute complex homolog	4.16E−06	Neuronal precursor
DCX	Doublecortin	1.84E−06	Neuronal precursor
FOXG1	Forkhead box G1	1.40E−02	Very rostal
EMX2	Empty spiracles homolog 2	3.64E−08	Forebrain
GLI3	GLI-Kruppel family member	3.34E−04	Dorsal forebrain
NEUROG2	Neurogenin 2	2.88E−03	Dorsal forebrain
LHX2	LIM/homeobox protein	3.16E−07	Dorsal forebrain
NR2F1	Nuclear receptor subfamily	1.29E−06	Ventral forebrain
MSX1	Homeobox msh-like 1	1.55E−04	Ventral midbrain
OTX1	Orthodenticle homolog 1	5.65E−07	Dorsal fore- and midbrain
OTX2	Orthodenticle homolog 2	3.29E−05	Dorsal fore- and midbrain
ATOH1	Math 1 atonal homolog	1.41E−02	Hindbrain
NCAN	Neurocan	2.69E−03	Neuronal adhesion and neurite growth during development
STX1A	Syntaxin 1A	2.37E−05	Synapse-associated
SYP	Synaptophysin	1.44E−02	Synaptic vesicle-associated
MAPT	Microtuble-associated protein tau	3.62E−06	Alzheimer-associated
TH	Tyrosine hydroxylase	1.71E−02	Dopaminergic neurons
TUBB3	Tubulin beta 3	3.45E−03	Cytoskeleton protein

For references, see (Kuegler et al. 2010)

When analyzing gene expression in unsupervised SOM (self-organizing map) analysis, three distinct clusters of gene expression emerged (Suppl. 3b). Of 4,140 genes regulated with high significance (FDR, false discovery rate <0.005), 1,814 genes were upregulated. Within these genes, the top 3 gene ontology (GO) categories were ‘nervous system development’, ‘neuron differentiation’, and ‘cell proliferation in forebrain’, indicative of robust differentiation along the neural lineage (Table 2).

**Table 2:** Top 10 GO categories in cluster 1 (upregulated genes)

GO category	Enrichment Score	Enrichment *p* value	% genes that are present	Genes in list/genes in GO	GO ID #
Nervous system development	16.8	5.0E−08	15.5	59/381	7399
Neuron differentiation	12.1	5.5E−06	24.3	18/74	30182
Cell proliferation in forebrain	11.3	1.3E−05	83.3	5/6	21846
Cilium	9.3	8.7E−05	18.4	21/114	5929
Negative chemotaxis	9.2	1.0E−04	62.5	5/8	50919
Multicellular organismal development	9.1	1.1E−04	10.8	97/900	7275
Synaptosome	9.0	1.2E−04	19.0	19/100	19717
Inner ear morphogenesis	8.8	1.5E−04	25.0	12/48	42472
Central nervous system development	8.4	2.2E−04	18.8	18/96	7417
Wnt receptor signaling pathway, calcium modulating pathway	8.4	2.3E−04	33.3	8/24	7223

### Expression of neural and neuronal markers during differentiation within neurospheres

**Fig. 2:**
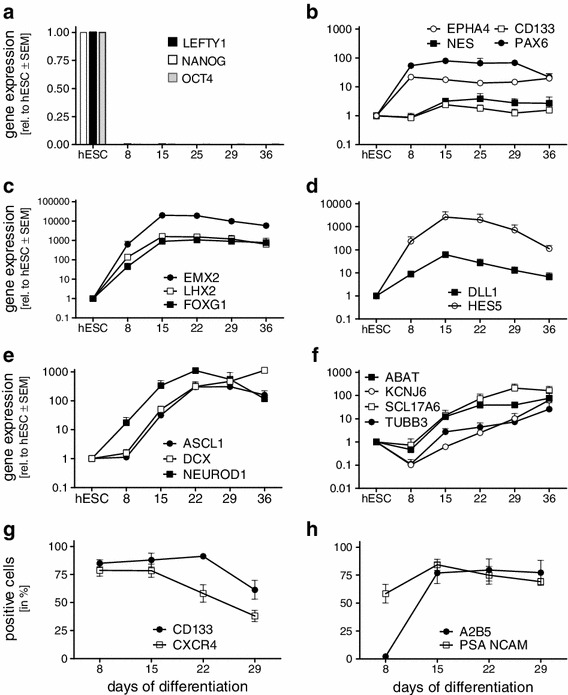
Marker expression during differentiation of hESCs to neural cells. hESCs were differentiated as in Fig. 1c and analyzed for marker expression. **a**–**f** At indicated time points, mRNA was extracted, gene expression quantified by quantitative RT-PCR and expressed as mean values normalized to expression in hESCs, set arbitrarily to 1. Mean values ± SEM, *n* = 4. **g**, **h** Expression of surface marker proteins measured by flow cytometry. Neurospheres were processed to single cells on indicated days and stained. Expression was assessed in viable cells and expressed as percentage of all viable cells. Mean values ± SEM, *n* = 6

Next, expression of markers specific for the different stages of neural differentiation was verified by quantitative RT-PCR. Pluripotency-associated genes such as NANOG and LEFTY1 became undetectable by d8 while POU5F1 decreased significantly, to remain expressed at very low levels (Fig. 2a). The neural stem cell markers EPHA4 and PAX6 peaked on day 8 of differentiation and remained expressed throughout the following 28 days of suspension culture, while CD133 and NES peaked between 7 and 14 days of suspension culture (d15–25) (Fig. 2b). Increasing expression of regional cortical patterning genes such as EMX2, LHX2, FOXG1, and PAX6 underlined the emergence of central nervous system (CNS) precursors (Fig. 2b, c). The neural progenitor markers HES5 and DLL1 both peaked on d15 and decreased thereafter (Fig. 2b), confirming the transcriptome data (Suppl. Fig. 3c). Increasing expression of neuronal precursor cell markers (ASCL1, DCX, and NEUROD1) and neuronal cell markers (ABAT, TUBB3, KCNJ6, and SLC17A6) strongly suggested the emergence of neuronal precursor cells and further differentiation to more mature neurons (Fig. 2e, f).

Surface expression of the neural progenitor cell markers CXCR4 and CD133 was found highest from d8 to d15, with peak expression of 78 ± 6 % and 88 ± 6 % of cells, respectively (Fig. 2g). A2B5, expressed on glial progenitors increased rapidly during the first week of suspension culture, to plateau around 80 ± 8 % of cells on day 22. PSA-NCAM, a marker for neuronal precursors, was already expressed on 58 ± 8 % of the cells on d8, to peak on d15 with 84 ± 5 % of positive cells, indicating the presence of a high proportion of neuronal precursor cells within the neurospheres (Fig. 2h).

### Sensitivity of the system to the developmental neurotoxicant methylmercury

**Fig. 3:**
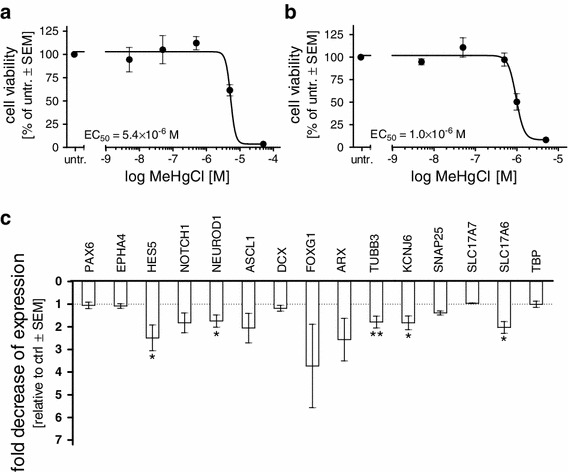
Acute and chronic toxicity of methylmercury during differentiation within neurospheres. Differentiating neurospheres were exposed on d11 to increasing doses of methylmercury. After **a** 48 h and **b** 18 days, cell viability was measured with resazurin reduction assay and expressed relative to untreated (untr.) control cells. **c** Cells were exposed 18 days to 50 nM methylmercury, mRNA extracted, and gene expression measured by quantitative RT-PCR. Expression was calculated as fold change compared to untreated (untr.) control which was arbitrarily set to 1. Data are mean ± SEM (*n* = 3), *p < 0.005, **p < 0.01)

Methylmercury is among the few chemicals that are known to elicit DNT in humans (Grandjean et al. 2006). We and others have shown that ESC-derived neural differentiation systems can detect toxic effects of methylmercury on marker gene expression already at low, non-cytotoxic concentrations (Stummann et al. 2009; Zimmer et al. 2011b, 2012). First, we assessed acute methylmercury toxicity to delineate the non-cytotoxic range. Cells were exposed three days after initiation of neurosphere cultures (d11) for 48 h and an EC_50_ value of 5.4 μM was measured (Fig. 3a). This was comparable to EC_50_ values obtained in HeLa and HEK cells (data not shown), indicating that the system, despite its relatively compact 3-D structure, responds similarly to the adherent cell lines.

Next, for DNT testing, starting on day 3 of suspension culture (d11), we exposed the neurospheres for 18 days. We measured an EC_50_ value of 1 μM (Fig. 3b). To test whether the presence of methylmercury had an impact on the differentiation process of the cells within the neurospheres, we investigated changes in gene expression after 18 days of exposure. Cells were exposed to the non-cytotoxic concentration of 50 nM, 1 log below the lowest non-cytotoxic concentration measured (500 nM). We chose a set of markers covering the entire neuronal differentiation process, including markers for neural precursors (PAX6, EPHA4), early and late neuronal precursors (HES5, NEUROD1, ASCL1, DCX), genes crucial for neuronal patterning (NOTCH1, FOXG1), as well as neuronal markers (ARX, TUBB3, KCNJ6, SNAP25, SLC17A6, SLC17A7). Analysis of gene expression by quantitative RT-PCR revealed significant decreases in gene expression in the neuronal precursor marker genes HES5 and NEUROD1 and the neuronal marker genes TUBB3, KCNJ6 (GIRK2), and SLC17A6 (VGLUT2), suggesting that during the differentiation to neural cells, the neurosphere model sensitively reacts to non-cytotoxic concentrations of methylmercury (Fig. 3c).

### Sensitivity of the system to toxicity of copper oxide nanoparticles (CuO-NPs)

CuO-NPs are highly toxic nanoparticles eliciting oxidative stress and DNA damage already at low concentrations (Bondarenko et al. 2012). To test whether the neurosphere model could detect NP toxicity, we exposed the neurospheres to CuO-NPs on d11 for 48 h. As exposure at higher concentrations of NPs interfered with resazurin reduction measurements, as observed by us and others, we measured intracellular ATP content as an alternative endpoint for cytotoxicity. The EC_50_ value was 35 μg/ml (Suppl. 7), comparable to the EC_50_ of 20 μg/ml measured in adherent cell lines such as A549 cells (Karlsson et al. 2008). However, as CuO-NPs release toxic copper ions over time, they are not an appropriate validation control to assess the long-term exposure effects in DNT measurements. Furthermore, we observed significant aggregation of CuO-NPs in culture medium which precluded an accurate determination of concentration-dependent toxicity of non-aggregated NPs. We, therefore, opted to use well-defined polyethylene NPs (PE-NPs), synthesized in-house under defined conditions.

### Assessment of acute toxicity of chemically inert polyethylene nanoparticles (PE-NPs)

**Fig. 4:**
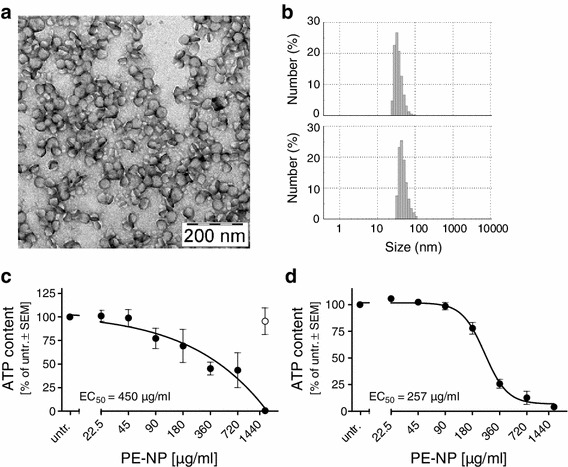
Acute toxicity of polyethylene nanoparticles (PE-NPs). Toxicity of PE-NPs was tested in HeLa cells and human neurons (LUHMES). **a** Transmission electron microscopy (TEM) image of PE-NPs, stained with phosphotungstic acid to increase contrast. **b** Dynamic light scattering (DLS) size distribution by number of PE-NPs in water (*upper panel*) and culture medium (*lower panel*). **c** Intracellular ATP content after incubation of HeLa cells with indicated concentrations of PE-NPs (*black circles*) for 48 h, expressed as proportion ± SEM of untreated (untr.) cells, *n* = 3. *Open circle data point* represents solvent control. **d** Intracellular ATP content after incubation of human neurons (LUHMES cells) with PE-NPs for 48 h, expressed as proportion ± SEM of untreated cells, *n* = 2–5

Polyethylene is an innocuous polymer considered to be biologically and chemically inert under physiological conditions. Moreover, it allows the synthesis of NPs with a defined and narrow size distribution and with the capacity to stay non-aggregated in culture medium. PE-NPs thus allow for the investigation of biological effects of biopersistent nanoparticles upon chronic exposure. PE-NPs were prepared in an aqueous microemulsion process, generating narrowly dispersed anisotropic nanocrystals with number average particle sizes of 33 nm (Fig. 4a, b) that could be maintained in aqueous dispersion in a stable non-aggregated state for prolonged periods of time (>3 months). No aggregation of PE-NPs occurred in cell culture medium (Fig. 4b, lower panel). To better visualize the uptake of PE-NPs, we incorporated a fluorophore during the synthesis process.

To determine the toxicity range, acute toxicity was measured after 48 h in HeLa cells, with an EC_50_ value of 450 μg/ml (Fig. 4c). To exclude toxicity of potential synthesis by-products in the PE-NP aqueous dispersion, toxicity of ultra-filtered PE-NP dispersion (devoid of PE-NPs, i.e., ‘solvent’ control) was also tested, and no changes in ATP content in cells treated with PE-NP-free dispersion were observed (Fig. 4c, open circle). As toxicity to HeLa cells may not reflect toxicity to neural cells, we also tested acute toxicity on mature dopaminergic human neurons (Schildknecht et al. 2009). LUHMES cells were incubated for 48 h with increasing concentrations of PE-NPs, and an EC_50_ value of 257 μg/ml was determined (Fig. 4d).

### Incorporation of PE-NPs into neurospheres

**Fig. 5:**
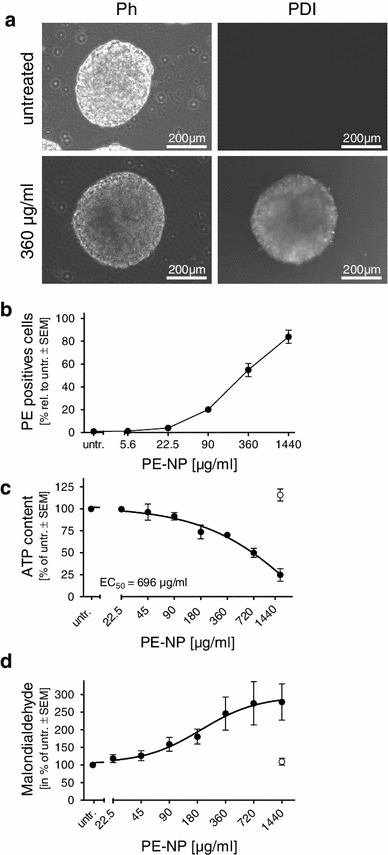
Incorporation into neurospheres and acute toxicity of PE-NPs. Differentiating neurospheres were exposed on d11 to PE-NPs for 48 h. **a** Neurospheres were exposed to 360 μg/ml of fluorescently labeled PE-NPs, and incorporation was monitored by fluorescence microscopy. **b** After 48 h, single-cell suspension was prepared and proportion of fluorescent cells measured by flow cytometry. Mean values ± SEM, *n* = 3. Measurement of intracellular ATP content (**c**) and malondialdehyde (**d**) after 48 h of exposure to PE-NP (*black circles*). Solvent control is indicated by *open circle*. Mean values ± SEM (*n* = 3). *Ph* phase contrast, *PDI* perylene diimide

Neurospheres were incubated on d11 with increasing concentrations of fluorescence-labeled PE-NPs, and incorporation was analyzed by fluorescence imaging and flow cytometry. PE-NPs were readily incorporated into the neurospheres after 24 h (data not shown) and were easily detected under the microscope at a concentration of 360 μg/ml at 48 h (Fig. 5a). Analysis of single-cell suspensions by flow cytometry revealed that despite the 3-D structure of the neurospheres, the PE-NPs had penetrated deep into the neurosphere and incorporated into most of the cells, with 84 ± 6 % of cells positive at the highest concentration used (Fig. 5b). Dose dependency of incorporation was reflected by increase in cytotoxicity, as assessed by ATP content measurements, with an acute EC_50_ value of 696 μg/ml (Fig. 5c). Similar to other nanoparticles, an increase in oxidative stress, that is, an increase in malondialdehyde, was measured after 48 h of exposure (Fig. 5d).

### Long-term toxicity of PE-NPs to developing neural cells

**Fig. 6:**
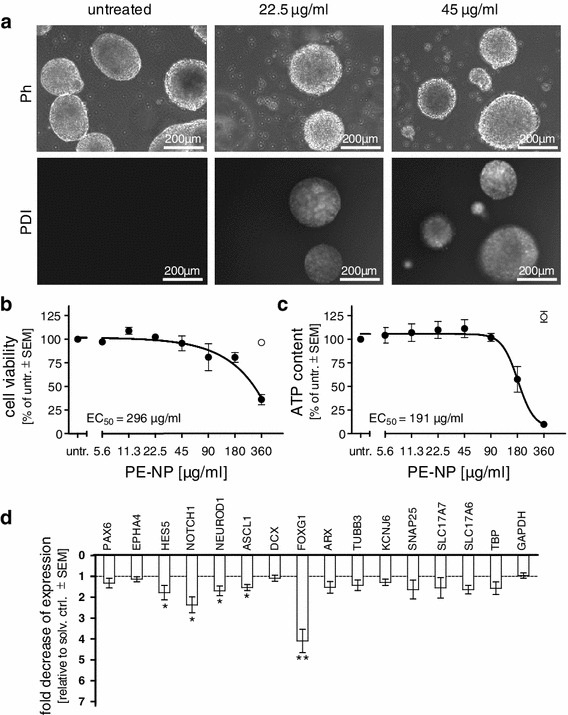
Chronic toxicity of PE-NPs in developing neurospheres. Differentiating neurospheres were exposed to PE-NP from d11 to d29. **a** Phase contrast and fluorescence images on d29. **b** Cell viability was measured with resazurin reduction assay on d29 and expressed as percent ± SEM of untreated (untr.) cells. *Open circle* solvent control (*n* = 2–5). **c** Cells were harvested on d29 and intracellular ATP content measured and expressed as percent ± SEM of untreated (untr.) cells. *Open circle* solvent control, *n* = 3. **d** Cells were exposed 18 days to 22.5 μg/ml of PE-NPs, mRNA extracted, and gene expression measured by qPCR. Expression was calculated as fold change compared to solvent (solv.) control which was arbitrarily set to 1. Mean values ± SEM (*n* = 3), *p < 0.005, **p < 0.01). *Ph* phase contrast, *PDI* perylene diimide

Neurospheres were exposed from d11 to PE-NPs for 18 days to determine the cytotoxic range. After 18 days, PE-NPs accumulation could be observed at 22.5 μg/ml (Fig. 6a). Cell viability measurements revealed EC_50_ values of 296 and 191 μg/ml, respectively, when resazurin reduction (Fig. 6b) or ATP content (Fig. 6c) were used as endpoints.

Next, we determined the impact of PE-NPs on the differentiation process and on the maturation of neural precursor cells more precisely. As we noted a slight toxicity of the filtered PE-NPs suspension (‘solvent’, free of PE-NPs) in longer exposures when assessing gene expression, we added this control in all following experiments. Neurospheres were exposed to 22.5 μg/ml of PE-NPs (or equivalent amounts of ‘solvent’), a concentration that was not cytotoxic. After 18 days of exposure, we measured the expression of a focused set of marker genes by quantitative RT-PCR. Notably, we observed a significant decrease in expressions of HES5, NOTCH1, FOXG1, NEUROD1 and ASCL1 (Fig. 6d). PAX6 expression, however, was not affected, suggesting that PE-NP toxicity had an impact on later progenitor populations. Unlike after methylmercury exposure, none of the tested later neuronal markers were significantly affected. Interestingly, no increase in gene expression was observed in any of the markers analyzed.

## Discussion

To our knowledge, the effects of engineered NPs on human neural development have not been investigated. A few human studies have documented the adverse health effects following exposure to environmental NPs, such as combustion-derived NPs in diesel exhausts, suggesting effects on inflammation, the cardiovascular system, and brain activity (Hesterberg et al. 2010). In prenatally exposed mice, engineered NPs have been detected, with ensuing neurobehavioral changes and impact on gene expression patterns in the brain (Yamashita et al. 2011). Such findings strongly suggest that NPs could, similar to methylmercury, trigger DNT in humans.

First in vitro reports investigating nanotoxicity during embryonic development are emerging. Park et al. (2009) reported the inhibition of differentiation by silica NPs in the embryonic stem cell test (EST), particularly of smaller-sized NPs, at concentrations below the cytotoxic range. The EST is currently the only ESC-based in vitro test system validated for regulatory use. A study by Pietroiusti et al. (2011) showed correlation of EST results with in vivo testing when assessing the toxicity of low-dose single-wall carbon nanotubes. However, as the EST uses murine ESCs, it may not reliably detect human-specific toxicity (Krewski et al. 2010). In its original form, it failed to predict the teratogenicity of thalidomide (Marx-Stoelting et al. 2009). Moreover, the EST tests impact on cardiac and not neural development and is thus not suitable to test for DNT. Similar to other reports of focused neural differentiation from hESCs, we, therefore, developed a system with targeted differentiation to neural cells (Colleoni et al. 2011, 2012; Stummann et al. 2009).

Transcriptome analyses in the here described new model revealed downregulation of pluripotency genes (Suppl. 5) and upregulation of key markers of neural development over time (Table 1). The differentiation out of PAX6^+^ CNS precursors and upregulation with high significance of several forebrain markers supports maturation toward CNS cells. Genes such as HOXA2 (rostral hindbrain), HOXB1 (rostral hindbrain), HOXB4 (caudal hindbrain), and HOXB6 (spinal cord) were not upregulated. Other genes upregulated with high significance during differentiation were represented in neural development-associated gene ontology (GO) categories such as ‘nervous system development,’ ‘neuron differentiation,’ and ‘cell proliferation in forebrain’ (Table 2). Overall, the transcriptome data suggest a predominantly forebrain-like development.

Among the top 30 regulated genes, 21 genes were upregulated. All upregulated genes, except 6 of unknown function, were associated with neural development and function (Suppl. 4), indicating a robust differentiation along neural lineage. Neural stem cell markers such as PAX6, EPHA4, and KCNN3 were strongly regulated. The same was observed for genes with a role in neural/neuronal development such as DMRT3, RSPO1, GPMA6, EVI1/PRDM3, ENKUR, ABHD14A, and GUCY1B3. Genes associated with disease such as MMRN1 (familial parkinsonism), ABAT (autism), and MAP6 (depression and schizophrenia-like symptoms) were also upregulated (for gene functions and references see Suppl. 4).

Analysis by quantitative RT-PCR confirmed the expression patterns observed in transcriptome analyses. Emergence of neuronal precursor cells was corroborated by the presence of a high percentage of PSA-NCAM protein-expressing cells. PSA-NCAM is the polysialylated form of neural cell adhesion molecule (NCAM) and is expressed on neuronal precursor cells in association with migrating phenotype, corroborating the increased expression of DCX, a marker for migrating neuroblasts (Quartu et al. 2008).

The known DNT substance, methylmercury, was used to test the performance of the developed 3-D model. Acute cytotoxicity was similar to that observed in standard cell lines such as HeLa cells, and long-term exposure resulted in an EC_50_ comparable to values measured by others in a hESC-derived neural differentiation systems (Stummann et al. 2009). When assessing the effects of 50 nM methylmercury exposure on the expression of neural marker genes, we detected an effect on the expression of the NOTCH target gene HES5. NOTCH1 itself was, however, not decreased significantly. Similar to Stummann et al., we also detected a decrease in the expression of the early neuronal precursor marker NEUROD1, but no significant reduction in NEUROD1 target genes ASCL1, DCX, and DLL1 (data not shown), suggesting a loss of certain subpopulations of neuronal precursor rather than a mere block of the NEUROD1 pathway. Contrary to Stummann et al., however, who detected no impact on neuronal genes at non-cytotoxic concentrations, we detected an effect on the expression of the neuronal genes TUBB3, KCNJ6, an ion channel modulating neuronal excitability, expressed in dopaminergic neurons (Reyes et al. 2012), and SLC17A6 (vesicular glutamate transporter). Other neuronal genes such as ARX, SNAP25, and SLC17A7 were not significantly affected.

Overall, our data suggest the selective loss of subpopulations of neuronal progenitors and possibly a defect in maturation toward neuronal phenotype in the presence of methylmercury. It will now be of interest to study the effects of methylmercury in more detail in this system to possibly delineate more precise mechanisms of methylmercury toxicity during neural development. TUBB3, for example, was also affected by methylmercury exposure in our murine DNT model (Zimmer et al. 2011b). Mutations in the TUBB3 gene result in so-called TUBB3 syndromes, which are more or less severe, depending on the exact type of mutation. Similar to methylmercury-induced poisoning, the spectrum of nervous system malformations includes spasticity, cognitive and behavioral impairments, and progressive peripheral sensorimotor axonal degeneration (Grandjean and Herz 2011; Tischfield and Engle 2010).

For DNT testing with NPs, we synthesized polyethylene NPs (PE-NPs) that remained non-aggregated in aqueous solution and cell culture medium over prolonged periods of time. PE-NPs were studied here as a model for surfactant stabilized, shape-persistent NPs composed of a hydrophobic chemically inert material. Similar to other materials where the bulk form is innocuous and the nanoparticulate forms toxic, we observed cytotoxicity of the PE-NPs both in adherent cells and neurospheres within 48 h (Figs. 4, 5, 6). PE-NPs penetrated deep into the 3-D neurosphere structure, and similar to a majority of NPs, elicited oxidative stress in the cells, and a dose-dependent cytotoxicity (Fig. 5b, c, d).

A key finding in this study was that at a concentration as low as 22.5 μg/ml of PE-NPs, we measured altered gene expression in the 3-D model. This concentration was four times lower than the lowest non-cytotoxic concentration measured. At this concentration, neural differentiation, as measured by changes in the expression of genes important for neurodevelopment, was perturbed. NOTCH pathway genes NOTCH1 and HES5, as well as downstream targets such as NEUROD1 and ASCL1 (both markers for neuronal precursor cells), were reduced in expression. NOTCH pathway plays a key role in both embryonic neural development and adult brain plasticity in animal models (Lasky and Wu 2005). NOTCH1 and NOTCH2 knockout mice die around E11, and HES5 gene-deleted mice have a 30–40 % reduction in Müller glial cells (Yoon and Gaiano 2005). NEUROD1 knockout mice develop a severe neurological phenotype (Miyata et al. 1999). ASCL1 is an important regulator of neurogenesis in the ventral telencephalon, and gene-deleted mice have multiple defects in neurogenesis in the ventral telencephalon, including a severe loss of neurons (Casarosa et al. 1999; Castro et al. 2011). Interestingly, expression of FOXG1, a brain-specific transcriptional repressor essential for the early development of the telencephalon, while not significantly affected by methylmercury treatment, was also reduced by PE-NP exposure. FOXG1 mutant mice die at birth with dramatic hypoplasia of the cerebral hemispheres, particularly of the ventral telencephalon (Xuan et al. 1995). Moreover, FOXG1 mutations are responsible for the congenital variant of Rett syndrome which, among other symptoms, includes microcephaly (Mencarelli et al. 2010).

Our data thus point to a potential impact of PE-NPs on progenitor cells and neuronal development, and furthermore suggest that NPs may affect the complex process of telencephalon differentiation.

Similar to other reports (Colleoni et al. 2011, 2012; Stummann et al. 2009) using targeted neural differentiation of hESCs to model human neural development and to assess neural-specific impact of toxicants, we developed here a 3-D model to assess nanotoxicity to neural cell differentiation and showed impact on the expression of genes crucial to neural development. However, it is unclear at this point, how a reduction in the expression of these crucial and early neurodevelopmental genes would impact in vivo neural development and elicit DNT. At this point, not many studies have investigated the impact of NPs on neural development in animal models. However, the few that have suggest that even innocuous NP such as TiO_2_-NPs reach the brain of the pups and have an impact on the development of the nervous system, with alterations in the dopaminergic system in the prefrontal cortex and moderate neurobehavioral alterations (Takeda et al. 2009; Hougaard et al. 2010; Takahashi et al. 2010). It was also shown that the NPs elicited changes in gene expression in the brains of the exposed pups (Shimizu et al. 2009). To our knowledge, no previous human-specific in vitro study has investigated the impact of NPs on neural differentiation. As a proof-of-principle, we have shown that our 3-D neurosphere model detects the known DNT compound, methylmercury, with good sensitivity and that expression of neurodevelopmental genes was affected upon NP exposure. Whether the used polyethylene NPs would affect developmental processes other than neural development is unclear at this point and could be tested in a humanized version of the EST. Testing of silica NPs and SWCNT in the original EST suggests NPs could be toxic to embryonic development in general (Park et al. 2009; Pietroiusti et al. 2011).

## Electronic supplementary material

Below is the link to the electronic supplementary material.

Supplementary material 1 (DOCX 1838 kb)
