# Supplementary material for: A 3-dimensional human embryonic stem cell (hESC)-derived model to detect developmental neurotoxicity of nanoparticles
Source: Arch Toxicol. 2012 Dec 2;87(4):721–33. doi: 10.1007/s00204-012-0984-2 (PMC3604581; doi:10.1007/s00204-012-0984-2)
Supplement: Supplementary file 1 — Supplementary material 1 (DOCX 1838 kb) [file 204_2012_984_MOESM1_ESM.docx]

SUPPLEMENTS

A 3-dimensional human embryonic stem cell (hESC)-derived model
to detect developmental neurotoxicity of nanoparticles

Archives of Toxicology

Lisa Hoelting^1,2^, Benjamin Scheinhardt^2,3^, Olesja Bondarenko^4^, Stefan Schildknecht^1^, Marion Kapitza^1^, Vivek Tanavde^5,6^, Betty Tan^5^, Qian Yi Lee^5^, Stefan Mecking^2,3^, Marcel Leist^1,2^, Suzanne Kadereit^1,2^

^1^Department of Biology, University of Konstanz, Germany, ^2^Konstanz Research School Chemical Biology, University of Konstanz, Konstanz, Germany, ^3^Department of Chemistry, University of Konstanz, Germany, ^4^Laboratory of Molecular Genetics, National Institute of Chemical Physics and Biophysics, Tallinn, Estonia, ^5^Bioinformatics Institute Agency for Science Technology and Research (A*STAR), Singapore, ^6^Institute for Medical Biology, A*STAR, Singapore

Corresponding author: suzanne.kadereit@uni-konstanz.de

Supplement 1. Primers used for qPCR.

| Name | Accession no. | Forward sequence | Reverse sequence |
| --- | --- | --- | --- |
| ABAT | NM_000663.4 | 5’-AGGAGGAGGCCCGCTGTCTG-3’ | 5’-CCAGAACTTGCCCGTGCAGCC-3’ |
| ARX | NM_139058.2 | 5’-CACTTGGCCCGACGGTTCTG-3’ | 5’-GGAGGAGGGGCTGCTGAAAC-3’ |
| ASCL1 | NM_004316.3 | 5’-GATGAGTAAGGTGGAGACACTGCG-3’ | 5’-CCGACGAGTAGGATGAGACCG-3’ |
| CD133 | NM_001145852.1 | 5’-CAGCTACTTGGCTCAGACTGG-3’ | 5’-AGGAAGGACTCGTTGCTGGTG-3’ |
| DCX | NM_001195553.1 | 5’- GCGAAATTTTTCAGGACCAC-3’ | 5’-CACAGAAGCCATCAAACTGG-3’ |
| DLL1 | NM_005618.3 | 5’-CAGGTGCCATGGAGACAGCC-3’ | 5’-GTGGGGAGCGTGGGGAGAAA-3’ |
| EMX2 | NM_004098.3 | 5’-CCAAGGGAACGACACTAGCC-3’ | 5’-CCATACTTTTACCTGAGTTTCCGTG-3’ |
| EPHA4 | NM_004438.3 | 5’-CCGTGGAGAGAGCCTTGTAA -3’ | 5’-GCCAAAAATGTACTGTGGGG -3’ |
| FOXG1 | NM_005249.3 | 5’-AGAAGAACGGCAAGTACGAGA-3’ | 5’-TGTTGAGGGACAGATTGTGGC-3’ |
| GAPDH | NM_001256799.1 | 5’-ATGGAGAAGGCTGGGGCTCA-3’ | 5’-AGTGATGGCATGGACTGTGGTCAT-3’ |
| HES5 | NM_001010926 | 5‘-TTG GAG TTG GGC TGG TG-3‘ | 5’-CCC AAA GAG AAA AAC CGA-3‘ |
| KCNJ6 | NM_002240.2 | 5’-TTCCTTCCCTCGCCATCCGT-3’ | 5’-CCACTGGGCTTTCGACGTCC-3’ |
| LEFTY1 | NM_020997.3 | 5’-AATGTGTCATTGTTTACTTGTCCTGTC-3’ | 5’-CAGGTCTTAGGTCCAGAGTGGTG-3’ |
| LHX2 | NM_004789.3 | 5’-GCTACTACCTGCTGGCGGTG-3’ | 5’-TCACCATCTCCGAGGCCGAG-3’ |
| NANOG | NM_024865.2 | 5’-GGTGAAGACCTGGTTCCAGAAC-3’ | 5’-CATCCCTGGTGGTAGGAAGAGTAAAG-3’ |
| NESTIN | NM_006617.1 | 5’-GCACCTCAAGATGTCCCTCAGC-3’ | 5’-GGGAAGTTGGGCTCAGGACTG-3’ |
| NEUROD1 | NM_002500.3 | 5’-GGATGACGATCAAAAGCCCAA-3’ | 5’-GCGTCTTAGAATAGCAAGGCA-3’ |
| NOTCH1 | NM_017617.3 | 5’-GAGGCGTGGCAGACTATGC-3’ | 5’-CTTGTACTCCGTCAGCGTGA-3’ |
| POU5F1 | NM_001173531.1 | 5’-GCAAAGCAGAAACCCTCGTGC-3 | 5’-ACACTCGGACCACATCCTTCTCG-3’ |
| PAX6 | NM_000280 | 5‘-CCGCCTATGCCCAGCTTCAC-3‘ | 5‘-AAGTGGTGCCCGAGGTGCCC-3‘ |
| RPL13A | NM_012423.2 | 5’-GGTATGCTGCCCCACAAAACC-3’ | 5’-CTGTCACTGCCTGGTACTTCCA-3’ |
| SLC17A6 | NM_020346.2 | Qiagen catalogue #.: 330001PPH14888A |  |
| SLC17A7 | NM_020309.3 | 5’-CTGCCCGCGTCCACTATGGC-3’ | 5’-GCACAAGGACCCCGGCGAGG-3’ |
| SNAP25 | NM_003081 | 5’-CTGTCTTTCCTTCCCTCCCT-3’ | 5’-GGGTCAGTGACGGGTTTG-3’ |
| TBP | NM_001172085.1 | 5’-GGGCACCACTCCACTGTATC-3’ | 5’-GGGCACCACTCCACTGTATC-3’ |
| TUBB3 | NM_006086.3 | 5’-AACTACGTGGGCGACTCGGA-3’ | 5’-GTTGTTGCCGGCCCCACTCT-3’ |

**Supplement 2. Synthesis of polyethylene nanoparticles.**

Fluorescence labeled as well as unlabeled polyethylene nanocrystals were prepared in an aqueous catalytic microemulsion polymerization process. In brief, trans-cyclooctene was subjected to ring opening metathesis polymerization by Grubbs-Hoveyda second generation catalyst in an aqueous microemulsion (H_2_O, SDS, pentanol). The resulting dispersion was hydrogenated exhaustively to yield a PE-NP dispersion. Volume and number weighted particle size distributions and polydispersity indices were determined via dynamic light scattering (DLS) on a Malvern Nano-ZS ZEN 3600 particle sizer (173° back scattering). The autocorrelation function was analyzed using the Malvern dispersion technology software 6.20 algorithm. The anisotropic nanocrystals typically had a number average particle sizes of 33 nm and a typical polydispersity index of 0.1. Between batches, this average size varied by ± 2 nm.

Covalent fluorescence labeling of the particles was achieved using a labeled ruthenium-alkylidene catalyst. As fluorescence marker a perylene diimide dye (N-(2,6-Diisopropylphenyl)-N’-(3-vinyl-4-isopropoxyphenyl)-1,6,7,12-tetra[4-(1,1,3,3-tetramethylbutyl)phenoxy]-perylene-3,4,9,10-tetracarboxylic diimide, PDI) was used, for its high quantum yield and excellent photo stability. The as-obtained polyethylene dispersions were extensively dialyzed against deionized water for several days to reduce the high surfactant (sodium dodecyl sulfate, SDS) content necessary in this procedure to yield dispersions with a surfactant and polymer content of 0.19 ± 0.03 % and 1.21 ± 0.18 %, respectively, and a zeta potential of –33 ± 4 mV. A high surface tension of 67 ± 3 mN m^‑1^ indicated complete removal of free surfactant.

The quantum yield of the fluorescence labeled polyethylene dispersions typically was 65 %, with absorption and emission maxima of 568 nm and 604 nm, respectively (see figure).

**Absorption (blue) and emission (red) spectra of a fluorescence labeled polyethylene dispersion.**

#
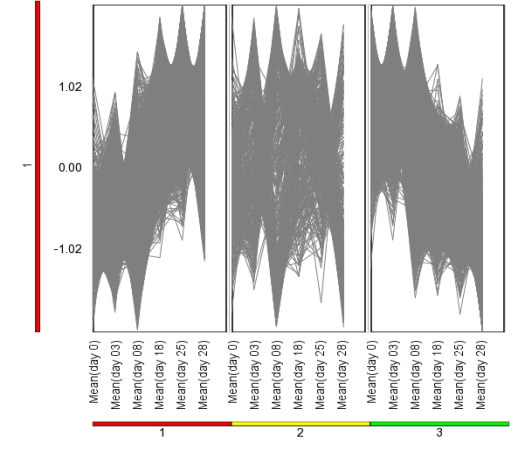


a

1 2 3

b

c

PCA mapping

(53.2%)

**Supplement 3. Transcriptome analysis during differentiation of hESCs to neural cells.** mRNA was extracted from hESCs and from differentiating cells on days 0, 5 (d0, d5, adherent cultures) and on days 15, 22 and 25 (d15, 22, 25, neurosphere cultures), hybridized in technical replicates to Illumina Sentrix HumanHT-12 BeadChip gene arrays and the data analyzed with Partek Genomic Suite. a) Principal component analysis, after grouping the technical replicates of the individual data sets (3 biological replicates for hESC-d8, 2 biological replicates for d15, singletons for d22, d25). b) Unsupervised self-organizing map (SOM) clustering analysis of the kinetic profiles of all significantly (FDR 0.005) regulated genes was performed, yielding 3 clusters. Cluster 1 (1814 genes) contained genes increasing in expression over time, cluster 2 (415 genes) contained genes with varying regulation patterns, and cluster 3 (1911 genes) contained genes that decreased over time. c) Expression profiles of individual marker genes graphed over time.

PC#2 17.3%

PC#3 11.5%


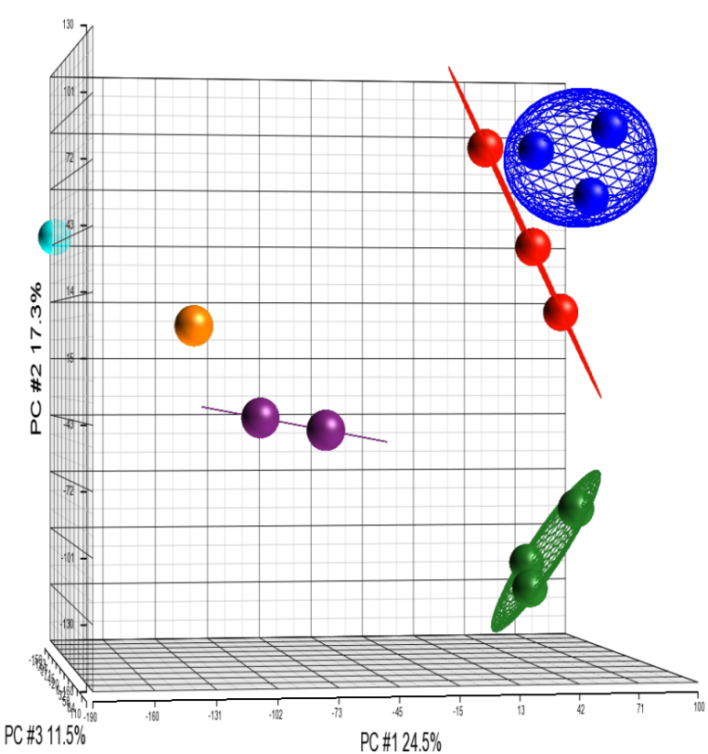


hESC

d0

d5

d15

d22

d25

PC#1 24.5%

Supplement 4. Top 30 regulated genes.

Mean Expression

hESC day05 day15 day22 day25 p value Function Ref.

**UP**

*FAM181A 5.0 4.9 10.7 10.2 6.9 3.63E-11 unknown function*DMRT3 4.6 5.4 10.4 10.5 11.2 2.73E-10 transcription factor, developing forebrain, neural tube and nasal placodes {Smith, 2002 #40}

PAX6 6.4 11.1 12.0 11.1 10.6 3.33E-10 very early transcription factor for central nervous system {Warren, 1999 #38}

RSPO1 5.0 5.1 10.4 10.6 9.7 2.25E-09 activator of the beta-catenin signaling cascade, ligand for FZD8 and LRP6 {Binnerts, 2007 #39}

EPHA4 8.1 11.9 10.2 9.9 8.8 4.60E-09 required for neural stem cell maintenance {Khodosevich, 2011 #67}

ABHD14A 7.2 7.8 8.6 9.4 7.1 4.89E-09 target of ZIC1, expressed in cerebellar granule neuronal precursors {Hoshino, 2003 #78}

*FAM181B 6.7 5.7 10.2 11.3 8.8 6.84E-09 unknown function*

KCNN3 5.5 5.6 6.7 7.3 6.4 1.03E-08 voltage-independent potassium channel expressed in embryonic NSC {Cai, 2004 #66}

*HS.421200 6.6 6.7 9.1 9.1 9.2 1.20E-08 unknown function*

*FAM116B 7.0 7.2 9.3 9.6 8.8 1.23E-08 unknown function*

TRH 5.0 5.0 11.9 13.0 12.9 1.32E-08 neurotransmitter/neuromodulator in CNS, PNS {Shibusawa, 2008 #42}

GPM6A 6.2 6.5 9.0 9.0 9.7 1.47E-08 neuronal membrane glycoprotein, role in neuronal differentiation {Mukobata, 2002 #79}

EVI1/PRDM3 5.5 6.7 11.1 10.7 8.4 1.50E-08 zinc finger transcription factor, role in development {Kania, 2005 #80}

MMRN1 5.5 5.4 10.3 10.5 7.7 1.58E-08 multimerin, associated with SNCA in familial Parkinsonism {Fuchs, 2007 #81}

GUCY1B3 6.0 6.8 8.4 7.8 7.7 1.61E-08 main receptor for NO, role in neurite outgrowth and synapse maturation {Lopez-Jimenez, 2009 #82}

*LOC338797 5.9 5.9 7.4 7.7 7.1 1.91E-08 unknown function*

*KIAA0895 6.3 7.4 9.2 9.1 9.0 2.03E-08 unknown function*ABAT 5.9 5.6 7.7 8.9 8.1 2.43E-08 4-aminobutyrate aminotransferase, GABA catabolism, association with autism {Barnby, 2005 #83}
CDH10 6.6 6.9 9.8 9.4 8.0 2.66E-08 blood-brain barrier adhesion molecule in CNS {Williams, 2005 #84}
ENKUR 5.8 5.7 7.1 7.2 5.7 3.07E-08 adaptor protein for TRPCs, channels with role in neuronal development {Sutton, 2004 #85;Tai, 2009 #86}

MAP6 6.2 6.3 9.2 9.6 9.3 3.32E-08 role in synaptic plasticity, depression and schizophrenia-like symptoms {Fournet, 2011 #87}

**DOWN**LECT1 11.4 8.3 6.4 6.2 6.2 3.12E-09 leukocyte cell derived chemotaxin, role in cartilage tissue {Hiraki, 1991 #88}

PNO1 10.2 9.6 8.1 7.7 7.6 5.86E-09 partner of NOB1, nuclear RNA binding protein, not expressed in brain {Zhou, 2004 #89}
*TMEM125 8.9 7.0 5.8 5.8 5.5 6.18E-09 transmembrane protein 125, unknown function*

CXCL5 7.9 6.3 6.0 5.9 6.0 1.51E-08 inflammatory chemokine {Mei, 2010 #91}

DPPA4 13.3 11.9 9.8 8.7 6.9 1.86E-08 pluripotency factor {Masaki, 2007 #90}

GAL 11.3 7.0 5.0 4.9 3.7 2.77E-08 small neuropeptide, cellular messenger within the CNS, PNS {Podlasz, 2012 #92}

TMPRSS2 7.8 6.9 6.0 6.0 5.9 3.00E-08 transmembrane serine protease, associated with prostate cancer {Afar, 2001 #93}

GRPR 9.6 7.1 6.2 6.0 6.1 3.24E-08 gastrin-releasing peptide receptor in neurons and glioma {Flores, 2010 #94}

LIN28 13.3 13.5 9.7 7.3 6.2 3.37E-08 hESC stemness marker, downregulated during ESC differentiation {Richards, 2004 #95}

CNS, central nervous system; NO, nitric oxide; NPC, neural progenitor cell; NSC, neural stem cell; PNS, peripheral nervous system

Supplement 5. Pluripotency genes regulated during differentiation.

|  | mean expression | | | | |  |  |
| --- | --- | --- | --- | --- | --- | --- | --- |
| Gene | hESC | day05 | day15 | day22 | day25 | p-value | regulation |
| DPPA4 | 13.3 | 11.9 | 9.8 | 8.7 | 6.9 | 1.86E-08 | down |
| LIN28 | 13.3 | 13.5 | 9.7 | 7.3 | 6.2 | 3.37E-08 | down |
| NANOG | 10.3 | 5.7 | 5.6 | 5.5 | 5.5 | 8.13E-08 | down |
| CD24 | 14.2 | 13.1 | 12.4 | 11.5 | 11.7 | 1.05E-07 | down |
| LEFTY1 | 13.2 | 6.3 | 6.4 | 6.2 | 7.5 | 1.35E-07 | down |
| POU5F1 | 12.1 | 8.2 | 5.9 | 5.7 | 5.9 | 6.59E-07 | down |
| DNMT3B | 11.8 | 9.1 | 8.1 | 7.8 | 6.6 | 9.93E-07 | down |
| JARID2 | 11.9 | 9.9 | 10.8 | 11.1 | 10.4 | 4.99E-05 | down |
| LEFTY2 | 11.9 | 6.4 | 6.4 | 6.4 | 6.2 | 7.00E-05 | down |
| UTF1 | 8.8 | 5.3 | 5.2 | 5.0 | 6.0 | 1.18E-04 | down |
| DPPA3 | 8.0 | 6.5 | 6.0 | 6.1 | 6.0 | 1.79E-03 | down |
| TERT | 6.1 | 5.2 | 5.4 | 5.3 | 5.1 | 7.47E-03 | down |

Supplement 8: Upregulation of neurodevelopmental genes during differentiation.


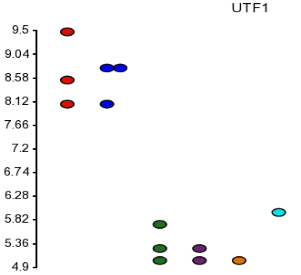


UTF1


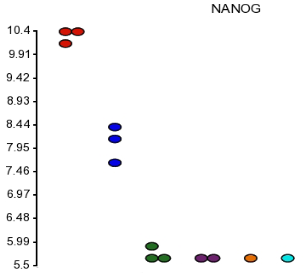


NANOG


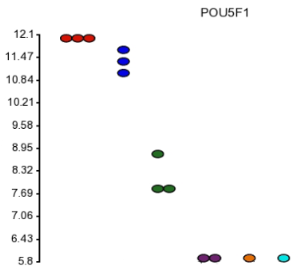


POU5F1


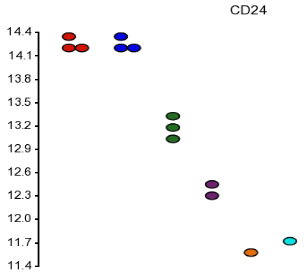


CD24


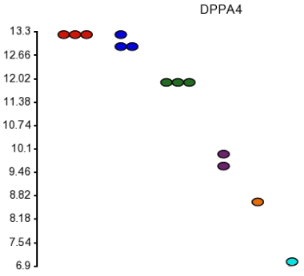


DPPA4


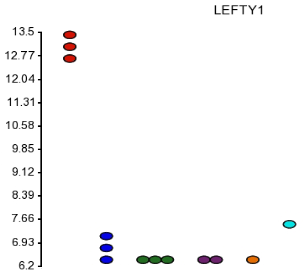


LEFTY1

**Supplement 6.** Gene array data was analyzed in Partek Genomic Suite and expression profiles of selected genes plotted as dot plots. a) Pluripotency genes. b) Neural stem and precursor cell markers and neuronal precursor markers. c) Neuronal markers.

PAX6 #3


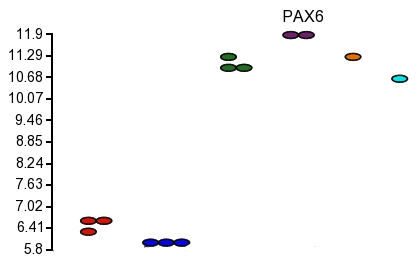


PAX6


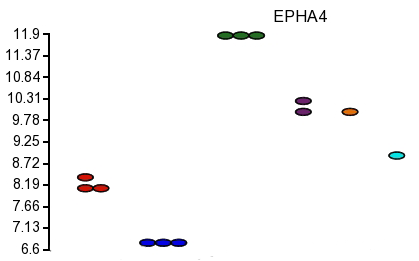


EPHA4


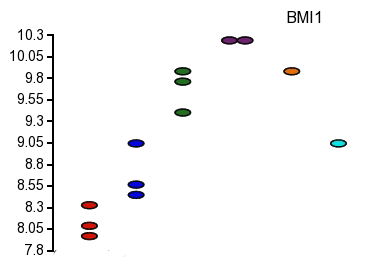


BMI1


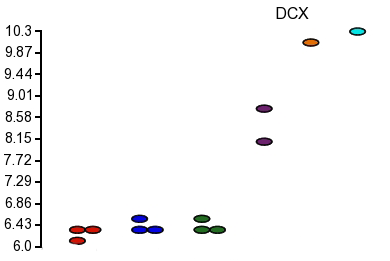


DCX


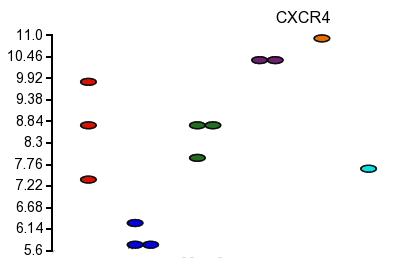


CXCR4


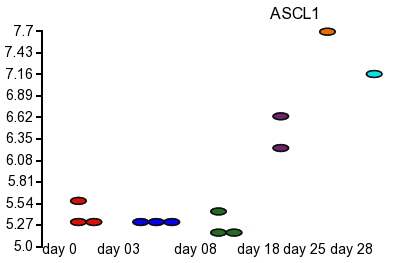


ASCL1


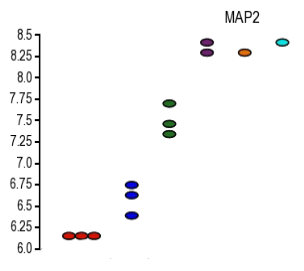


MAP2


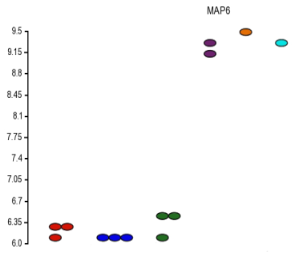


MAP6


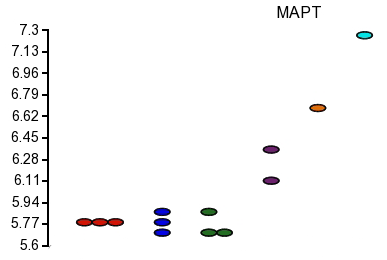


MAPT


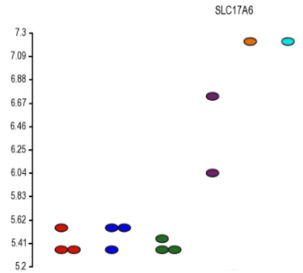


SLC17A6


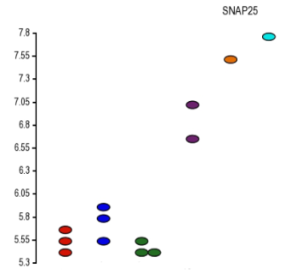


SNAP25


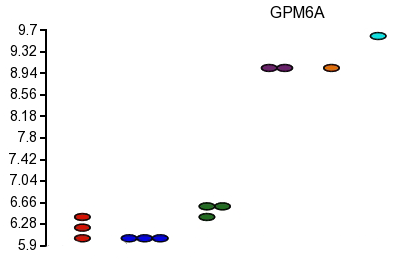


GPM6A

a

b

c

hESC

d0

d5

d15

d22

d25

**Supplement 7. Acute toxicity of copper oxide nanoparticles in neurospheres.** Neurospheres on d11 of differentiation were exposed to copper oxide nanoparticles (CuO-NPs, Sigma Aldrich, cat. #. 544868, Cu(II)oxide nanopowder, <50 nm particle size ) for 48 h and cell viability assessed by measurement of intracellular ATP content and expressed as percent of untreated (untr.) cells. Are graphed means ± SEM of 3-4 repeat experiments.

| Gene |  | Fold change  (hESC vs day 25) |  |
| --- | --- | --- | --- |
| LHX2 | LIM/homeobox protein | 113.1 |  |
| EMX2 | Empty spiracles homolog 2 | 87.8 |  |
| Hes5 | Hairy and enhancer of split 5 | 51.1 |  |
| NR2F1 | Nuclear receptor subfamily | 34.1 |  |
| OTX1 | Orthodenticie homolog 1 | 25 |  |
| DCX | Doublecortin | 18.6 |  |
| DLL1 | Delta-like 1 | 8.1 |  |
| MSX1 | Homeobox msh-like 1 | 6.8 |  |
| STX1A | Syntaxin 1A | 6 |  |
| NEUROG2 | Neurogenin 2 | 3.7 |  |
| GLI3 | GLI-Kruppel family member | 3.5 |  |
| Ascl1 | Mash1, achaete-scute complex homolog | 3.4 |  |
| MAPT | Microtuble-associated protein tau | 2.9 |  |
| TH | Tyrosine hydroxylase | 2.4 |  |
| TUBB3 | Tubulin beta 3 | 2.3 |  |
| FOXG1 | Forkhead box G1 | 2 |  |
| OTX2 | Orthodenticie homolog 2 | 1.6 |  |
| SYP | Synaptophysin | 1.4 |  |
| NCAN | Neurocan | 1.3 |  |
| ATOH1 | Math 1 atonal homolog | 1.2 |  |

Fold changes of the neurodevelopmental genes from Table 1.

Supplement 9: Predicted upstream regulators of genes regulated significantly during the differentiation process

| Upstream regulator | p-value | | | | | significantly regulated genes |
| --- | --- | --- | --- | --- | --- | --- |
|  | d0 | d5 | d15 | d22 | d25 |  |
| ASB9 |  | 1.1E-02 | 4.2E-02 |  | 4.2E-02 | CKB |
| CTNNB1 |  | 1.5E-02 |  |  |  | CDH11,CTNNA2,FGF9,GAD1,POU3F2 |
|  |  |  | 2.7E-03 |  |  | ALDH1A1,BCL2,CDH11,CTNNA2,EPHB3,FGF9,HTRA1,LMO2, MSX1,MSX2,PDE4B,POU3F2,RCN1 |
|  |  |  |  | 1.7E-03 |  | ALDH1A1,BCL2,CDH11,CTNNA2,ENO2,EPHB3,FGF9,HTRA1, IL10, LMO2,MSX1,MSX2,POU3F2,RCN1,SERPINA5 |
|  |  |  |  |  | 1.8E-02 | BCL2,CDH11,EPHB3,FGF9,HTRA1,IL10,LMO2,MSX1,MSX2, POU3F2,RCN1 |
| E2F3 |  | 3.0E-02 |  |  |  | BMI1,CCNA1 |
| E2F5 |  | 4.4E-03 |  |  |  | CCNA1,VEGFB |
| FLI1 | 2.8E-02 |  |  |  |  | ID2 |
| FOSL1 | 3.1E-02 |  |  |  |  | SPARC |
| FOXL2 | 9.7E-03 |  |  |  |  | RSPO3,SMAD6 |
| FOXP3 | 4.9E-02 |  |  |  |  | ID2 |
|  |  |  | 2.8E-02 | 4.4E-02 |  | ID2,MAF,PDCD4 |
| GLI1 | 2.9E-02 |  |  |  |  | CMBL,ID2 |
| HDAC7 |  | 2.3E-02 |  |  |  | RPRM |
| HIC1 | 8.1E-04 |  |  |  |  | CXCR7,ID4,SOX9 |
|  |  |  | 1.7E-04 | 1.5E-04 |  | CA2,CDKN1C,CDO1,CXCR7,ID4,RIN2,SNAP25,SOX9 |
|  |  |  |  |  |  | CA2,CDKN1C,CDO1,CXCR7,ID4,RIN2,SNAP25,SOX9 |
|  |  |  |  |  | 7.8E-04 | CA2,CDKN1C,CDO1,RIN2,SNAP25,SNAPC1,SOX9 |
| HMGA1 |  |  |  |  | 1.5E-02 | IDI1,INSIG1,KRT10,MVD |
| HOXA10 |  | 4.5E-02 |  |  |  | EMX2 |
| HOXA13 | 3.7E-02 |  |  |  |  | WNT5A |
| LYL1 | 3.4E-02 |  |  |  |  | ID4 |
| MBD1 | 4.9E-02 |  |  |  |  | PTPRM |
| MLL2 |  |  | 1.5E-02 |  | 1.5E-02 | CRIP2,ENO3,GPR56,NPR3 |
| MTA1 | 4.9E-02 |  |  |  |  | CXCR7 |
| MXI1 | 3.7E-02 |  |  |  |  | ID2 |
| MYBL1 |  |  | 4.2E-02 |  | 4.2E-02 | BCL2 |
| NANOG |  | 2.4E-02 |  |  |  | LHX2,OTX1,PAX6 |
|  |  |  | 1.9E-03 | 5.4E-03 |  | IER5L,LHX2,MEIS1,OTX1,PAX6,PDCD4,TBC1D3,ZFHX3 |
|  |  |  |  |  | 7.4E-03 | LHX2,MEIS1,OTX1,PAX6,PDCD4,TBC1D3,ZFHX3 |
| NEUROD1 |  |  | 4.2E-02 |  | 4.2E-02 | SLIT2 |
| NFIC | 9.3E-03 | 3.4E-02 |  |  |  | FABP7 |
| NFYA | 4.6E-02 |  |  |  |  | COL11A1 |
| NOTCH1 |  |  | 2.0E-02 |  |  | ASCL1,EFNB2,HEY1,NR2F2 |
|  |  |  |  | 7.4E-03 |  | ASCL1,EFNB2,ENO2,HEY1,NR2F2 |
| NOTCH2 | 1.9E-02 |  |  |  |  | HEY1 |
|  |  |  | 2.4E-02 | 3.3E-02 |  | ASCL1,HEY1 |
| NOTCH4 | 9.3E-03 |  |  |  |  | HEY1 |
| NRF1 |  | 3.4E-02 |  |  |  | CD47 |
| NRIP1 | 1.5E-02 |  |  |  |  | SMAD6 |
| PKNOX1 | 1.2E-02 | 4.5E-02 |  |  |  | FABP7 |
| POU5F1 |  | 3.5E-03 |  |  |  | LHX2,OTX1,PAX6 |
|  |  |  | 7.9E-04 | 1.9E-03 |  | IER5L,LHX2,MEIS1,OTX1,PAX6,ZFHX3 |
|  |  |  |  |  | 5.0E-03 | LHX2,MEIS1,OTX1,PAX6,ZFHX3 |
| PROX1 | 2.2E-02 |  |  |  |  | HEY1 |
| SIX1 |  | 1.1E-02 | 4.2E-02 |  | 4.2E-02 | CCNA1 |
| SMAD2 |  |  | 4.2E-02 |  |  | CCNG2,TIMP3 |
| SNAI1 | 4.3E-02 |  |  |  |  | SPARC |
| SNAPC2 | 3.1E-03 |  |  |  | 4.2E-02 | RN7SK |
| SNAPC4 | 3.1E-03 |  |  |  | 4.2E-02 | RN7SK |
| SOX2 |  | 4.3E-03 |  |  |  | LHX2,OTX1,PAX6 |
|  |  |  | 1.2E-03 | 2.8E-03 |  | IER5L,LHX2,MEIS1,OTX1,PAX6,ZFHX3 |
|  |  |  |  |  | 6.8E-03 | LHX2,MEIS1,OTX1,PAX6,ZFHX3 |
| SOX5 |  | 1.1E-02 | 4.2E-02 |  | 4.2E-02 | SPAG6 |
| SOX9 |  |  | 2.4E-02 | 3.3E-02 | 2.4E-02 | COL2A1,GAS7 |
| SPDEF | 1.2E-02 |  |  |  |  | COL5A2,WNT5A |
|  |  | 3.4E-04 |  |  |  | CDH11,COL5A2,LAMB2,LEF1,VEGFB |
|  |  |  | 3.5E-04 | 1.2E-03 | 3.4E-04 | CDH11,COL4A5,COL4A6,COL5A2,DKK3,LAMB2,LEF1,SMAD3 WNT5A |
| SRY | 6.2E-03 |  |  |  |  | SOX9 |
| STAT2 |  |  | 4.2E-02 |  | 4.2E-02 | IFI27,IFI6 |
| TWIST2 |  | 1.2E-02 |  |  |  | BMI1,ZEB2 |
| WWTR1 | 2.2E-02 |  |  |  |  | ID2 |
| ZNF217 |  | 1.5E-04 |  |  |  | CXXC4,GAD1,LMO3,PAX6,WNT5B,ZHX2 |
|  |  |  | 9.4E-03 |  |  | CXXC4,IGDCC3,LMO3,LYPD1,PAX6,RAB22A,SHC4,WNT5B |
|  |  |  |  | 8.3E-03 |  | CXXC4,IGDCC3,LMO3,LYPD1,PAX6,RAB22A,SEC14L2,SHC4, WNT5B |
|  |  |  |  |  | 2.7E-03 | CXXC4,IGDCC3,LMO3,LYPD1,PAK3,PAX6,RAB22A,WNT5B, ZHX2 |

The list of differentially expressed genes was imported into Ingenuity Pathways Analysis (IPA) to predict the upstream regulators of genes contained within the data set analyzed. The table shows the predicted transcription factors/regulators with their corresponding downstream target(s) that were significantly regulated at day 0, day 5, day 15, day 22 of differentiation.

| Supplement 10. Abbreviations | |  |
| --- | --- | --- |
|  |  | |
| 3-D | Three dimensional | |
| ATP | Adenosine-5'-triphosphate | |
| CNS | Central nervous system | |
| CuO-NPs | Copper oxide nanoparticles | |
| DLS | Dynamic light scattering | |
| DNT | Developmental neurotoxicity | |
| EC_50_ | Half maximal effective concentration | |
| EST | Embryonic stem cell test | |
| FDR | False discovery rate | |
| GO | Gene ontology | |
| hESC | Human embryonic stem cell | |
| LUHMES | Lund human mesencephalic | |
| mESC | Mouse embronic stem cell | |
| NEP | Neural epithelial progenitors | |
| NO | Nitric oxide | |
| NPC | Neural progenitor cell | |
| NPs | Nanoparticles | |
| NSC | Neural stem cell | |
| PCA | Principal component analysis | |
| PDI | Perylene diimide | |
| PE-NPs | Polyethylene nanoparticles | |
| Ph | Phase contrast | |
| PNS | Peripheral nervous system | |
| qPCR | Quantitative reverse transcriptase PCR | |
| ROS | Reactive oxygen species | |
| SOM | Self-organizing map | |
| TEM | Transmission electron microscopy | |
| TiO_2_ | Titanium dioxide | |
